# Bibliographical

**Published:** 1883-05

**Authors:** 


### Bibliographical.

*A Handbook of Materia-Medica and Therapeutics for Dentists and Dental Students;* by D. R. Stubblefield, A. M., M. D., D. D. S., Professor of Anatomy and Physiology in the Dental Department of Vanderbilt University.

The time when dentistry was but a mere mechanical art has long since passed away, and the dental student of to-day, if he has an ambition to become a peer among his fellows, must have a thorough knowledge, not only of anatomy and physiology, but of the various remedies which he will be called upon to prescribe. The most of the medical text-books contain matter which it is not absolutely essential that practitioners of dentistry should master. It is better that the average dentist should know well the properties and therapeutical value of such drugs as he will most frequently be required to use, rather than that he should attempt to gain a smattering of the whole medical pharmacopœia. To the attainment of this end, text-books, peculiarly adapted to his needs, are required. Heretofore the chief reliance has been upon the excellent materia-medica of Dr. Stocken. But this has not completely met all wants, and we were therefore prepared to receive a new work with favor.

An examination of Dr. Stubblefield's work has given us great pleasure, and already it has become almost indispensable as a book of ready reference. It is not entirely devoid of errors, but the concise system upon which it is written will commend it to every dental practitioner. The amount of information concerning every remedy which the dentist will be likely to use, that is crowded into so small a compass, is surprising, yet so perfect is the collocation that almost at a glance all the prominent characteristics may be seen.

The consideration of Chloral Hydrate, for instance, occupies nearly two pages. Within this space is given its officinal and common names, its symbol, its history, its method of manufacture, its physical characteristics, its antagonists, incompatibles and antidotes, its synergists, its action and uses, its therapeutical virtues and vices, a list of diseases and conditions in which it is especially valuable, the usual doses, and a number of written prescriptions. The same system is pursued with other remedies, and a very complete index enables the student readily to find any information of which he may be in search. We commend the book to every dentist who desires to practice intelligently. It may be procured of the author, at Nashville, Tenn.

*Some of the Opportunities, Responsibilities, and Encouragements of Life.* An address delivered before the Massachusetts Dental Society, at its eighteenth annual meeting, in Boston, December 14th, 1882.

By C. A. Brackett, D. M. D., of Newport, R. I.

Published by request of the society.

A pamphlet which every thinking man, whether professional or non-professional, would do well to read and seriously ponder over. Dr. Brackett is known as an accomplished writer, and nothing better than this has come from his pen.

We have received from the S. S. White Dental Mfg. Co., but too late for critical notice in this number, *Notes on Operative Dentistry*, by Marshall H. Webb, D. D. S. It will receive due attention next month.

